# The *Penefit* of Salience: Salient Accented, but Not Unaccented Words Reveal Accent Adaptation Effects

**DOI:** 10.3389/fpsyg.2016.00864

**Published:** 2016-06-07

**Authors:** Ann-Kathrin Grohe, Andrea Weber

**Affiliations:** Psycholinguistics and Applied Language Studies, English Department, Faculty of Humanities, University of TübingenGermany

**Keywords:** native accents, adaptation, eye-tracking, salience

## Abstract

In two eye-tracking experiments, the effects of salience in accent training and speech accentedness on spoken-word recognition were investigated. Salience was expected to increase a stimulus' prominence and therefore promote learning. A training-test paradigm was used on native German participants utilizing an artificial German accent. Salience was elicited by two different criteria: production and listening training as a subjective criterion and accented (Experiment 1) and canonical test words (Experiment 2) as an objective criterion. During training in Experiment 1, participants either read single German words out loud and deliberately devoiced initial voiced stop consonants (e.g., *Balken*—“beam” pronounced as ^*^Palken), or they listened to pre-recorded words with the same accent. In a subsequent eye-tracking experiment, looks to auditorily presented target words with the accent were analyzed. Participants from both training conditions fixated accented target words more often than a control group without training. Training was identical in Experiment 2, but during test, canonical German words that overlapped in onset with the accented words from training were presented as target words (e.g., *Palme*—“palm tree” overlapped in onset with the training word ^*^Palken) rather than accented words. This time, no training effect was observed; recognition of canonical word forms was not affected by having learned the accent. Therefore, accent learning was only visible when the accented test tokens in Experiment 1, which were not included in the test of Experiment 2, possessed sufficient salience based on the objective criterion “accent.” These effects were not modified by the subjective criterion of salience from the training modality.

## Introduction

Languages typically consist of a number of regional dialects—that is, native accents. In the southwestern German state of Baden-Württemberg, for example, one does not have to travel very far to encounter various native accents, as Spiekermann documented in 2008 (Spiekermann, 2008). This variation can pose a problem for non-locals. When non-locals hear a native accent for the first time, they often do not understand what is being said as easily as do locals, who are experienced with the regional varieties. Recent studies have indeed shown that listeners process accents in their native language more easily when they are familiar with the accents than when they are unfamiliar with them (e.g., Adank et al., 2009). Adaptation by non-locals to native accents is, however, possible. Adaptation has been found for longer periods of exposure to a novel accent (Evans and Iverson, 2007), but it can even be observed after just 4 min of listening to a new accent (Trude and Brown-Schmidt, 2012). This is also true for second language (L2) learners. Producing a new accent for only 7 min can facilitate subsequent accent understanding for L2 learners, even more so than listening to the accent does (Grohe and Weber, in press). The act of speech production arguably makes an accent more salient than listening to that accent does. Can the advantage of production experience also be observed in a listener's native language (L1)? Next to signal modality (production and listening), salience can also emerge from concrete properties of the speech signal itself. Acoustic distinctiveness of a speech signal can enhance its salience (e.g., Cho and Feldman, 2013). The present study used a training-test paradigm in German in which salience was induced by either a subjective or an objective criterion and looked at the role of salience in native accent adaptation. The subjective criterion was implemented through two different accent trainings (production and listening) and the objective criterion through the featuring of either accented (Experiment 1) or canonical (Experiment 2) target words during test in an eye-tracking study.

### Adaptation to native accents

Familiarity with a native accent facilitates accent processing. For example, listeners with extensive experience with the New York City (NYC) English accent show greater priming effects for words with the NYC-English-typical final r-dropping than listeners with limited experience (Sumner and Samuel, 2009). Similarly, Adank et al. (2009) found that only listeners who were familiar with both Southern Standard British English (SSBE) and Glaswegian English (GE) showed equal performance on both accent types in a sentence verification task. The familiarity advantage probably results from adaptation processes, as demonstrated by Evans and Iverson (2007). In their study, students who were originally from Northern England adapted to SSBE over the course of their university studies in Southern England, as shown through production and comprehension tasks. Processing advantages for participants' own accents over an unfamiliar accent were also found for French listeners (Floccia et al., 2006). In a lexical decision task, reaction times to items in long sentences were faster when sentences were presented in the participants' own accent (Northeastern French) than when they were presented in the unfamiliar Southern French accent. Furthermore, participants did not adapt to the unfamiliar accent during the course of the experiment (see also Floccia et al., 2009). Additionally, Adank and McQueen (2007) found no short-term adaption in a study with regionally-accented Dutch. In their study, Dutch participants who were not familiar with the Flemish accent had to make animacy decisions on single words spoken by two different speakers, one with a Flemish accent and one with the same accent as the participants. Then, participants were exposed to another speaker with the Flemish accent before having to repeat the animacy decision task. Decision times in the second animacy task were not faster than in the original task.

Short-term adaptation was, however, found by Trude and Brown-Schmidt (2012). Participants were first trained on a native English accent and then tested in an eye-tracking paradigm. During training, participants listened to scripted dialogs with an accented male speaker and an unaccented female speaker. The male speaker raised the /æ/ before /g/ to [eI], i.e., *bag* was pronounced /beIg/. During test, target words were either spoken by the male or the female speaker. When *back*, a word unaffected by the accent, was the target and *bag* the competitor, *bag* was ruled out more quickly as a candidate word for trials with the male speaker than it was with the female speaker. When *bake*, a word with /eI/ in its canonical form, was the target and *bag* acted as competitor, *bake* was fixated less often when it was spoken by the male. This effect, however, was less strongly pronounced, i.e., competitor inclusion was more difficult than competitor exclusion.

Specific properties of the tested accents could account for the missing effects of adaptation in the studies discussed above, but, more importantly, speaker-specificity can explain it too. In contrast to Adank and McQueen, who had different speakers with the same accent during exposure and test and did not find accent adaptation, Trude and Brown-Schmidt used the same accented speaker in both of their two experimental phases. Short-term adaptation to native-accented speech may therefore be rather speaker-specific. This problem has also been addressed in studies on foreign accent adaptation, with mixed results. Using a training test paradigm, Bradlow and Bent (2008) found that generalization of accent learning (Chinese-accented English) to new voices is only possible if the listener is exposed to multiple speakers during training (for similar findings see also Sidaras et al., 2009). Kraljic and Samuel (2007), on the other hand, found with L1 listeners that the nature of the tested material has an effect on whether perceptual learning can generalize to new speakers. They found generalization effects for plosives but not for fricatives.

### Adaptation with production

Speaker specificity raises the question of whether there is a way to make training more effective, i.e., allowing for generalization across speakers, and potentially rendering competitor inclusion more robust. This might be possible through production training. In a recent study by Grohe and Weber (in press), the production of a foreign accent in participants' L2 promoted adaptation to that accent in a subsequent lexical decision task. Participants first either listened to an English short story that featured the replacement of all dental fricatives (“th”) with /t/ (e.g., *theft* pronounced as ^*^ *teft*), or they read the same story aloud with the same substitutions. A control group had no accent training. Afterwards, all participants completed a lexical decision task on words with the th-substitutions. The production group accepted the accented words significantly more quickly than the control group did. The listening group produced only a weak training effect. When the same experiment was run with L1 participants, no training effect was observed. Referring to speaker effects, L1 participants in the production group produced the critical accent marker, but they were listening to an L2 speaker in the test phase. According to Pickering and Garrod (2013), listeners are more likely to refer to their own previous production experience if it is highly similar to the speaker they are listening to (e.g., in terms of sex, L1 background, dialect). Less similarity leads listeners to draw on their experience with others' speech. Since only the L2 participants in Grohe and Weber had the same L1 background as the recorded test speakers, speaker-listener similarity was smaller for L1 participants than for L2 participants.

Facilitatory effects of producing an accent were also found in an accent imitation study with L1 speakers of Dutch (Adank et al., 2010). Baese-Berk and Samuel (2016), however, found that imitating a newly learned L2 sound can even inhibit learning. In their study, participants had to imitate sounds from a sound continuum ranging from /$\underset{}{\underset{⎵}{\text{s}}}$ a/–/∫a/, which is arguably difficult for speakers to imitate correctly. A potential acoustic discrepancy between the sound prompt that was presented and the participants' productions may therefore have inhibited learning effects. A recent discrimination study with Danish vowels (Kartushina et al., 2015) supports this interpretation. In that study, production accuracy was increased by concrete feedback on productions, which in turn resulted in better sound discrimination performance after production than after listening training.

### Salience in adaptation

We now turn to the concept of salience, which can potentially explain both the results of accent adaptation and the advantage of produced compared to listened-to tokens. Salience has been generally defined as “the property of a linguistic item or feature that makes it in some way perceptually and cognitively prominent” (Kerswill and Williams, 2002, p. 81). An important question, however, is what exactly makes a linguistic item salient.

First, sociolinguistic research on salience suggests that an accent can increase a word's salience. As suggested by Trudgill (1986), the phonetic difference between two variant forms affects their salience; the greater the difference, the more a dialect speaker is aware of it. Phonetic distance can also be considered within the framework of distinctiveness which assumes that isolated, i.e., distinct, words are more salient than others during encoding—provoking additional processing and, therefore, better memory (McDaniel and Geraci, 2006, for a review). Geraci and Manzano (2010), for example, had participants study a list of semantically related words that also included a few semantically unrelated, i.e., distinct, words. In ensuing tests, more unrelated than related words were recalled. Accordingly, Siegel (2010) claims that salience requires a listener to notice a contrast between two linguistic tokens. In terms of phonetic variability, words that carry an accent are distinct from their unaccented counterparts and bear greater salience. Therefore, they can be learned more easily than unaccented words^1^. This was tested in a different memory study (Cho and Feldman, 2013). L1 English participants listened to either Dutch-accented or native-accented English words during a training phase. In a subsequent word recognition task, there was an advantage for Dutch-accented words.

Second, factors beyond linguistic or structural properties may also affect salience. For the case of dialect accommodation, Kerswill and Williams (2002) suggest intensity of dialect contact as one of several factors. Considering the findings on production effects on accent adaptation, we can extend the list of extra-linguistic factors toward cognitive mechanisms by introducing accent learning modality (production vs. listening) as an additional factor. Several studies have found an advantage of production over listening for dialect accommodation; this has been named the *production effect*. It predicts that overt production facilitates word recollection when compared with studying a word silently (MacLeod et al., 2010) and also when compared with listening to others producing a word aloud (MacLeod, 2011). It has been suggested that produced words are more easily recalled because they are more distinctive and therefore more salient. Distinctiveness results from listeners focusing more on their own than on others' productions, which are, in the sense of the embodiment hypothesis (for an overview: Glenberg, 2010), more embodied than others' productions.

Salience, as described above, has been further specified in sociolinguistic research. Referring to Schirmunski (1928) and Trudgill (1986), Auer et al. (1998) differentiate objective and subjective criteria of salience. For example, *articulatory distance* is described as an objective criterion and *perceptual distance* as its subjective counterpart. The two relate in that articulatory distance describes the magnitude which a linguistic token deviates acoustically from the canonical realization, whereas perceptual distance describes which way a listener perceives this distance. Based on this information, we can conclude that subjective criteria increase the salience of a stimulus, for example, due to regular practice, and the resulting cognitive pre-activation. Objective criteria refer to properties of a stimulus that itself attracts attention because of its distinct, physical characteristics. Under this view, the production effect relies on the presence of an objective criterion. A self-produced word can be physically more distinct compared to a word read silently or a word that is produced by others because these words were only tested in mixed lists, i.e., one participant had to listen to/silently read and produce words in the same session.

In summary, prior research has shown that native accents are more easily processed if they are familiar to a listener than if they are new. Short-term adaptation to native accents is possible, and production alone can positively affect foreign accent learning, at least in L2 learners. Both robust accent adaptation and the role of production in accent adaptation may be related to salience. The role and concrete nature of salience in learning accented vs. canonical words through different forms of training, however, is not yet clear.

### Present study

The present study takes a closer look at this issue by investigating subjective and objective criteria of salience separately, using modality and accent as criteria. In an exposure-test paradigm, German participants first underwent native accent training before adaptation was tested by a printed word eye-tracking task. A subjective criterion was established by having two different types of training (production and listening), while the objective criterion featured accented vs. canonical test words. Accented test words had their initial voiced bilabial or velar stop devoiced. In Experiment 1, accented words (^*^Palken for *Balken*—“beam”) were presented during training and test. In Experiment 2, the same accented words were presented during training (^*^Palken for *Balken*), but target items during test were canonical words that overlapped in onset with the trained accented word (***Pal****me*—“palm tree”). Old word pairs from the training phase as well as new word pairs that had not been included in the training list were tested. This manipulation was included to test generalization of learning, i.e., whether the accent is only learned for trained words or also for new accented words.

A subjective criterion of salience was tested by comparing effects of individuals' accent productions with that of listening during training. In contrast to MacLeod (2011), the current study did not manipulate training modality in mixed lists within participants but rather between participants. This permits the comparison of the magnitude of salience based on a subjective criterion of the production modality with that of the listening modality. Individual participants are exclusively trained with one modality. If producing an item in fact constitutes a subjective criterion for salience compared to listening to an item, production training with that item would result in greater salience than listening training.

An objective criterion of salience, on the other hand, was manipulated by the presence of both accented and canonical test tokens. In Experiment 1, the presentation of accented words assigned salience to the test tokens due to their great degree of inherent distinctiveness. Effects of accent as an objective criterion have been previously shown (Cho and Feldman, 2013), but with a memory experiment in which generalization effects were not examined. In the learning phase, the accented words were embedded in a list of filler target words that featured no particular accent marker. This made the accented words distinct from the fillers. Contrarily, in the present Experiment 2, the canonical test words were expected to be less salient. Experiment 2 tested whether the salience inherent in the learned accent can modify the processing of words that include the accent target sound in their canonical form. A difference in learning effects can be reflected in the activation differences of canonical target words starting with the manipulated accent's target sound. Learning that *Balken* is pronounced as ^*^Palken potentially increases lexical competition for the canonical *Palme*, which, in turn, slows down recognition of *Palme*. This is based on Trude and Brown-Schmidt (2012), who found that accent learning can imply the inclusion of new competitors. In the present study, *Balken* could be included as a new competitor for *Palme* after training, resulting in fewer target looks to *Palme*.

The pattern of target and competitor activation is especially important during the segmental overlap of target and competitor words. Referring to the principles of an abstract mental lexicon, we assume that accent learning is based on learning pre-lexical rules. When hearing ^*^Palken in Experiment 1, successful word recognition requires the application of a specific accent rule (/b/ → /p/). If the accent rule is learned robustly during training, it is applied by default as soon as the auditory input potentially matches the accent, i.e., from initial /p/ presentation onward. When, in an eye-tracking experiment, the display includes *PALME* and *BALKEN* and ^*^Palken is the auditory target, both *PALME* and *BALKEN* should be fixated from word onset until disambiguation (including /pal/). Only after disambiguation should *BALKEN* be fixated more often than *PALME*. If the accent rule is not learned robustly enough, the candidates that require the rule (*BALKEN*) have a weaker activation than those that do not require the rule (*PALME*). Consequently, during the overlapping word portion, *PALME* will still be more strongly activated than *BALKEN*, and *BALKEN* will only be preferred after disambiguation.

Successful recognition of a canonical word (*Palme*), as in Experiment 2, does not require accent rule application. However, successful accent learning should result in increased competitor activation of words with a /b/ in initial position. This increase in competitor activation might adversely affect canonical target activation. The rule should be applied by default as soon as the auditory input potentially matches the accent, i.e., also when *Palme* is presented. Having *PALME* and *BALKEN* on the visual display, both words should be equally fixated during /pal/. Only after disambiguation should *PALME* be preferred. The same predictions as above emerge if the accent rule is not learned strongly enough—the candidates that require the rule (*BALKEN*) are activated less strongly than canonical words (*PALME*). Consequently, *PALME* will be more strongly activated than *BALKEN* even from the beginning of word processing.

The accent in the present study is an artificial accent that centers on one specific phonological accent marker and therefore must be differentiated from a dialect. Participants and pre-recorded speakers are not L2 speakers, and all used standard German pronunciation in the experimental context. “Standard” here means that the pre-recorded speakers did not have a noticeable dialect that could allocate them to a specific region in Germany, and the participants' speech did not include specific local (e.g., Swabian) accent properties during the experiment. The tested accent affected German stop consonants and has, to our knowledge, not been documented as an existing native accent of German. It refers to the lenis/fortis-contrast in German bilabial and velar plosives (/b, p/ and /g, k/). In Standard German, fortis plosives are always aspirated in word initial position, while lenis plosives are never aspirated (Jessen, 1998; Kleiner and Knöbl, 2015). Our accent neutralized this contrast, i.e., lenis velar and bilabial stops were aspirated (/g/ pronounced [k^h^] and /b/ pronounced [p^h^]: *Gitter* pronounced ^*^Kitter, and *Balken* pronounced ^*^Palken). The accented sound was always aspirated, similar to the canonically fortis stops. For simplification, we refer to aspirated, fortis plosives (*Palme*) as “voiceless” and to the lenis plosives with the additional aspiration in the accented version as “devoiced” (^*^P*alken*).

We opted for an accent with a target sound that is included in the German sound inventory (Kohler, 1999). This makes it easy to produce for native German participants and promises relatively stable acoustic properties of the target sounds across participants. The accent under investigation has to be differentiated from middle-Bavarian dialects where bilabial, alveolar, or velar plosives are not realized with an aspiration contrast before /r, l, n, m/; they are always voiceless and unaspirated and therefore neutralize with their lenis counterpart, e.g., *Preiselbeeren*—“cranberries” pronounced ^*^Breiselbeeren (Moosmüller and Ringen, 2004). Likewise, in Austrian German, the fortis plosives /p/ and /t/ are not aspirated (Siebs et al., 1969), e.g., *Pinsel*—“brush” pronounced ^*^Binsel. In contrast, the accent presented in this study neutralized all bilabial plosives to [p^h^] and all velar plosives to [k^h^]. Since the accent tested in our study describes a voicing shift in the opposing direction of existing native German accents, we can assume that none of our participants had had experience with the accent. This ensured the observation of only laboratory-specific training effects.

We predict that accent training will result in accent learning effects. The training modality can determine the amount of salience based on one subjective criterion. This would be in line with prior findings where producing rather than listening to a word resulted in better memory (MacLeod, 2011). Salience that relies on an objective criterion of the target token is expected to affect looking patterns such that the learned accent affects processing of highly salient, accented devoiced tokens more than that of canonical voiceless tokens.

## Experiment 1

Experiment 1 tested if salience can result from training as subjective criterion. Critical test words had a native accent and were assumed to be highly salient based on the objective criterion “accent.” During training, native German participants either read aloud or listened to single German words that had their initial /b, g/ devoiced to /p, k/, e.g., *Balken* pronounced as ^*^Palken, while the control group had no training.

In the test phase of the experiment, participants accomplished the printed word variant (McQueen and Viebahn, 2007; Weber et al., 2007) of the eye-tracking task (Allopenna et al., 1998). Participants saw four printed words in their canonical spelling (including a target, a competitor, and two distractors) on a computer screen and were auditorily instructed to click on a target word while their eye movements were recorded. They listened to devoiced words (^*^Palken) and had to click on a visual display that included the target word (*BALKEN*) and a competitor (*PALME*). The competitor allows the investigation of whether activation of the devoiced token can be as strong as activation of voiceless word forms without an accent. The proportion of target fixations was measured and compared between the production, listening, and control groups.

### Participants

Seventy-four native German speaking female students (19–30 years, mean age = 23.8, *SD* = 2.7; 5 left-handed) from the University of Tübingen participated for a small monetary remuneration. Only women were tested in order to account for the fact that the recordings were exclusively made by female speakers. German was their only mother tongue^2^, they did not suffer from any hearing disorders and had normal or corrected-to-normal vision. Two participants were excluded due to unsuccessful calibration, resulting in the collection of data from 72 participants (26 production group, 22 listening group, and 24 control group).

### Materials

#### Words during the test phase

We presented 92 word quadruplets during test, each containing four German nouns. Twenty-eight quadruplets were based on critical word pairs; 64 quadruplets were based on filler word pairs. The 28 critical word pairs were each composed of a target word with an initial voiced stop and a competitor word starting with the corresponding voiceless stop. Only target words were presented auditorily during the experiment. Fourteen had a bilabial onset (e.g., target *BALKEN* “beam”—competitor *PALME* “palm tree”), and 14 had a velar onset (e.g., target *GITTER* “grid”—competitor *KITTEL* “tunic”). We opted for plosives, because it has been shown that perceptual learning of plosives can generalize across speakers (Kraljic and Samuel, 2007), arguably because they contain hardly no talker-specific information in comparison to fricatives, for example. This was important because participants in the training groups were trained with a different voice than was heard during test. Voiced stops occurred only in the initial position of target words. The initial stop consonant was always followed by a vowel^3^. Apart from the initial consonant, target and competitor overlapped in at least two segments. When the target words were presented auditorily, the initial voiceless plosives were devoiced (*Balken* was pronounced as ^*^Palken), resulting in overlapping word onsets of target and competitor for at least three segments. Auditory words with the native accent (^*^Palken) were never existing words of German (see Table A1 for target-competitor pairs).

Mean log-frequencies of target words were 0.61 per million for velar stop words, 0.85 for bilabial stop words, and of competitors 0.67 for velar stop words, and 0.88 for bilabial stop words according to the CELEX word form dictionary (Baayen et al., 1995). In order to form quadruplets, each of the 28 critical target-competitor pairs was paired with two semantically unrelated distractor words that matched in frequency with the target-competitor pair. Distractor words never had a stop in initial position but could contain stop consonants in other word positions.

The 64 filler word pairs also had a target and a competitor. There were 8 targets with initial /k/, 8 with initial /p/, 16 with initial /t/, and 32 targets with no initial stop in onset position (the “no-stop targets”). For the total of 32 targets with initial /k/, /p/, and /t/, half of the competitor word onsets overlapped with the target word onset by at least three segments, and half were phonologically unrelated. Two phonologically and semantically unrelated distractors were added to each target-competitor pair. The 32 no-stop targets were paired with competitors that also did not have stops in initial position. However, half of them overlapped in onset with the target for at least two segments (e.g., target *Seife* “soap”—competitor *Seite* “side/page”). There were four types of distractors for the 32 no-stop target-competitor pairs, each containing eight distractor pairs. The bilabial (b/p), velar (g/k), and alveolar (d/t) distractor pairs followed the same prerequisites as the corresponding critical target-competitor pairs. As they were not presented auditorily, stop+consonant onsets (e.g., *Brosche* “brooch”—*Prospekt* “brochure/leaflet”) were allowed. The fourth group had two semantically and phonologically unrelated initial sounds that were never stops.

Altogether, the test included 92 critical trials and four practice trials. Half of the critical targets and their corresponding competitors had been included in the preceding training phase, and half were new to participants. Likewise, half of the targets not starting with a stop (other-group) were new to participants and half were familiar from the training. Every participant had her own experimental list, each starting with the same four practice trials. Filler and critical trials were equally distributed across the lists, and a critical trial was always followed by at least one filler trial. There were never more than two old and not more than five new trials in a row. The various filler conditions were equally distributed across the lists.

#### Words during the training phase

Seventy-two single words from the above described target-competitor pairs were used for training. They included half of the devoiced targets (7 targets with bilabial onset, e.g., ^*^Palken, and 7 targets with velar onset) and their respective competitor (*Palme* for target ^*^Palken). The devoiced and voiceless items were included twice in the training list, resulting in 28 devoiced and 28 voiceless trials. Additionally, 16 filler targets from the no-stop targets were included, resulting in 72 training trials in total. Training trials with the same initial sound did not occur more than twice in a row, and each devoiced item was followed by at least one canonical item.

#### Recordings of test and training tokens

All tokens used for training and test were recorded by two female native speakers of Standard German without a noticeable regional accent (speaker A: 23 years; speaker B: 28 years). The speakers did not differ significantly in F0-range or speaking rate, and the authors judged their pronunciation to be comparable. Two different speakers were recorded to have different voices for both the training and test phases in the listening group. This permitted constant conditions across the training groups because the production condition always involved a different speaker during the training (the participant) and the test (the pre-recorded talker). Nevertheless, speaker-listener similarity was granted by having participants and pre-recorded speakers with the same sex and L1 background in the test phase. Acoustic differences between the training and test tokens are held as small as possible. Moreover, it can be tested whether speaker specific effects, as observed by Trude and Brown-Schmidt (2012), can generalize to new speakers of the same sex (both female).

Recordings were carried out in a sound proof cabin with an Olympus LS-11 sound recorder (44.1 kHz; 16-bit). Every target word was recorded in the context of the carrier sentence *Klicken Sie jetzt auf* —“Now click on.” The devoiced target words (^*^Palken) and the voiceless words (*Palme*) were all recorded naturally, that is, the speakers were explicitly instructed to pronounce the /b/ in *Balken* the way they would pronounce the /p/ in *Palme*. The best exemplar of the carrier sentence was chosen for each voice, and the duration of the carrier sentence was matched between both voices. Then, the carrier sentence was added to each target word recording.

### Procedure

An *SR-Research Eyelink 1000* set-up was used for data collection with a sampling rate of 1 kHz, and the experiment was programmed with *Experiment Builder* (SR Research Ltd., Canada). Before the experiment started, the dominant eye of each participant was determined. Then, participants were seated in front of a computer screen and placed their chin on a chin rest. They were brought to a position in which they could stay comfortably for the duration of the experiment (~30 min). The eye-tracker was calibrated, then written instructions were shown on the screen. Participants had as much time as they needed to read them and initiated the experiment with a mouse click.

#### Training

The same training list was presented to each participant from the two training groups, while the control group received no training. The training tokens were presented either visually and auditorily (listening group) or visually only (production group). The listening group first saw a fixation cross for 1000 ms, then the orthographic transcript of the training word (black Arial font, size 24) appeared in the center of the screen. It corresponded to German spelling rules (*BALKEN*). The initial letter that corresponded to the devoiced sound was colored red. Five hundred milliseconds later, the training word was played (^*^Palken). Participants listened to the single words (devoiced, voiceless, and fillers) through noise-canceling headphones (Sennheiser HD 215 II) and at the same time fixated the transcript on the screen. There was a 2000 ms inter-trial interval. Participants in the listening group were explicitly told to listen attentively to the words and to be aware of the speaker's accent while fixating the orthographic version of the words. Witteman et al. (2013) have shown that a single word context is sufficient for listeners to learn an accent. In their cross-modal priming task participants without previous accent exposure had increased priming effects in the second half of the experimental list compared to the first half.

The production group did not wear headphones during the training. They saw the same orthographic transcript of the words on the screen and had to read every single word out loud while their productions were recorded. Participants were asked to pronounce the initial red letter “B” as /p/ and the initial red letter “G” as /k/. Before every single trial, there was a fixation cross for 1000 ms, and then the word was shown for 3500 ms (accounting for the timing in the listening condition: 500 ms before the sound + 1000 ms mean word duration + 2000 ms pause). The next trial would then start. Between training and test, the written instructions for the eye-tracking task were shown on the screen. The production group had about 5 s to put on their headphones, and the listening group waited for 5 s until the initiation of the test phase. Overall, the training took about 7 min for each participant.

#### Test phase

**Figure 1:**
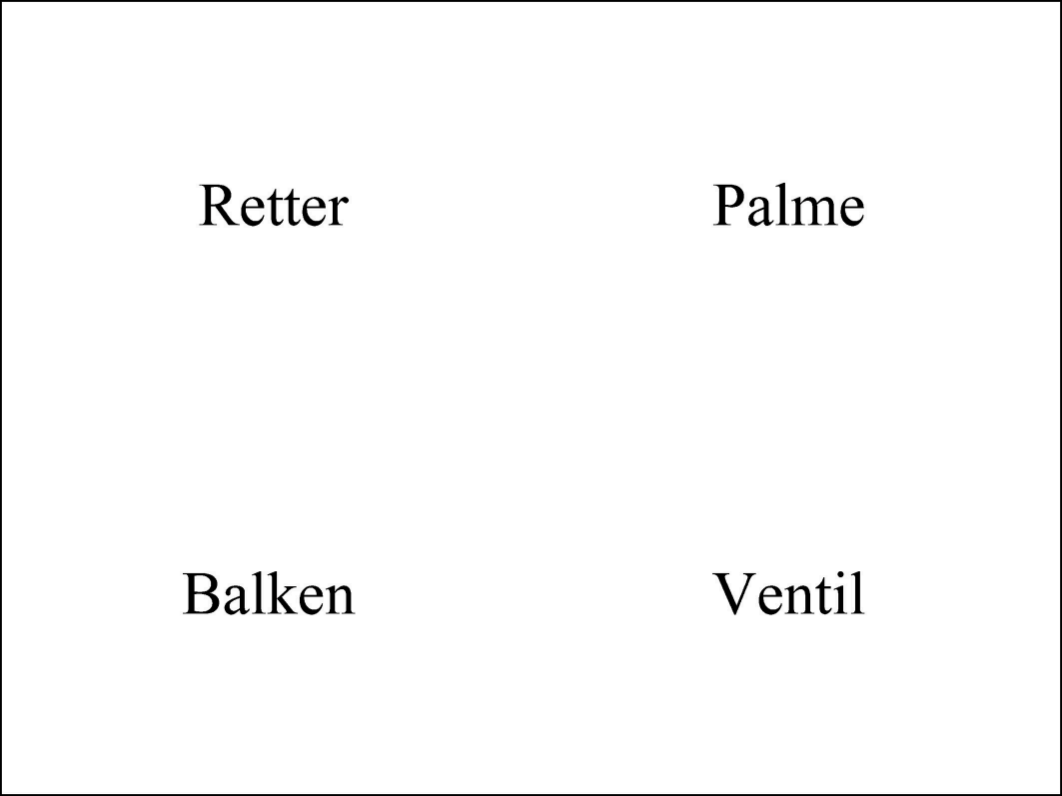
**Example display of a test trial in Experiments 1 and 2**. In Experiment 1, *BALKEN* was the target and *PALME* the competitor. In Experiment 2, *PALME* was the target, and *BALKEN* the competitor. *RETTER* and *VENTIL* were distractors in both experiments.

The test phase started with four practice trials. A fixation cross preceded each trial for 1000 ms, then four printed words from a word quadruplet were shown on the screen for 500 ms. The words were printed in black Times New Roman font, size 34 on a screen with a white background. Screen resolution was 1024 × 768 pixels, and the words were distributed across four different positions (256 × 576, 768 × 576, 256 × 192, and 768 × 192 pixels), see Figure 1. Display positions of target and competitor were pseudo-randomized, and the target never appeared in the same display position more than three times in a row. The mouse cursor (represented by a small circle) was located in the center of the screen at the beginning of each trial. Then the carrier sentence (about 1300 ms) followed by the target word was played auditorily. Participants clicked on the target word with the mouse. Visually, participants saw the target word in its correct spelling (*BALKEN*); auditorily, it had the same accent as presented during the training phase (^*^Palken). A small fixation circle appeared on the screen after every six trials to initiate an automatic drift correction in the calibration of the eye-tracker. The experiment concluded with a language background questionnaire based on the *LEAP-Q* (Marian et al., 2007).

### Analysis and results

During training, the production group performed the instructed accent quite well. The experimenter decided based on perceptual judgments whether the critical training tokens were devoiced as communicated in the instructions. Every instance where the devoicing was not clearly perceivable was documented and subsequently validated by means of acoustic measurements of the recordings. On average, only 0.7 out of 28 critical trials were not devoiced as instructed. The proportion of correct clicks on the target during the test phase was 94.3% (equally distributed across the training groups). However, five participants did not see the mouse cursor due to technical problems. We extracted fixation reports with the software *Data Viewer* (SR Research) and then further processed the data with the software *R* (R development core team, 2015). The data from each participant's dominant eye was used to determine the coordinates and timing of fixations. Only fixations that fell within a cell of one of the four interest areas—target, competitor, and two distractors—were analyzed (exclusion of 3.4% of the data). The interest areas each had a cell size of 472 × 344 pixels with a distance of 40 pixels between vertical cells and 60 pixels between horizontal cells. Saccades (20.8% of the data) were not added to fixation times. We then analyzed the fixations for the four interest areas in 20-ms steps in a time window from 0 to 1000 ms after target word onset. The dependent variable “target” indicated whether in the respective 20-ms step a participant fixated the target; “competitor” indicated a competitor fixation, and “distractor” a distractor fixation. This resulted in three variables with binary values. Target and competitor fixation proportions were calculated with the empirical logit function. The plotted fixation proportions were inspected visually to determine the critical time window to which linear mixed effects regression models (Baayen et al., 2008; Bates et al., 2015) were then applied. For each analysis we built an individual, best fitting model that included a particular choice of fixed and random factors. Random effect structure included random intercepts for participants and items as well as those random slopes that significantly improved the model fit as tested by likelihood ratio tests. Significance of factors was indicated by *t* > |2|. Corresponding *p*-values, as reported in the text below, were determined with likelihood ratio tests. As fixed effects, we considered training (production vs. listening vs. no training), familiarity (old, i.e., included in the training, vs. new), sound condition (bilabial vs. velar word initial sound), and speaker (speaker A vs. speaker B). Proportion of target fixations was the dependent variable.

#### Descriptive analysis

**Figure 2:**
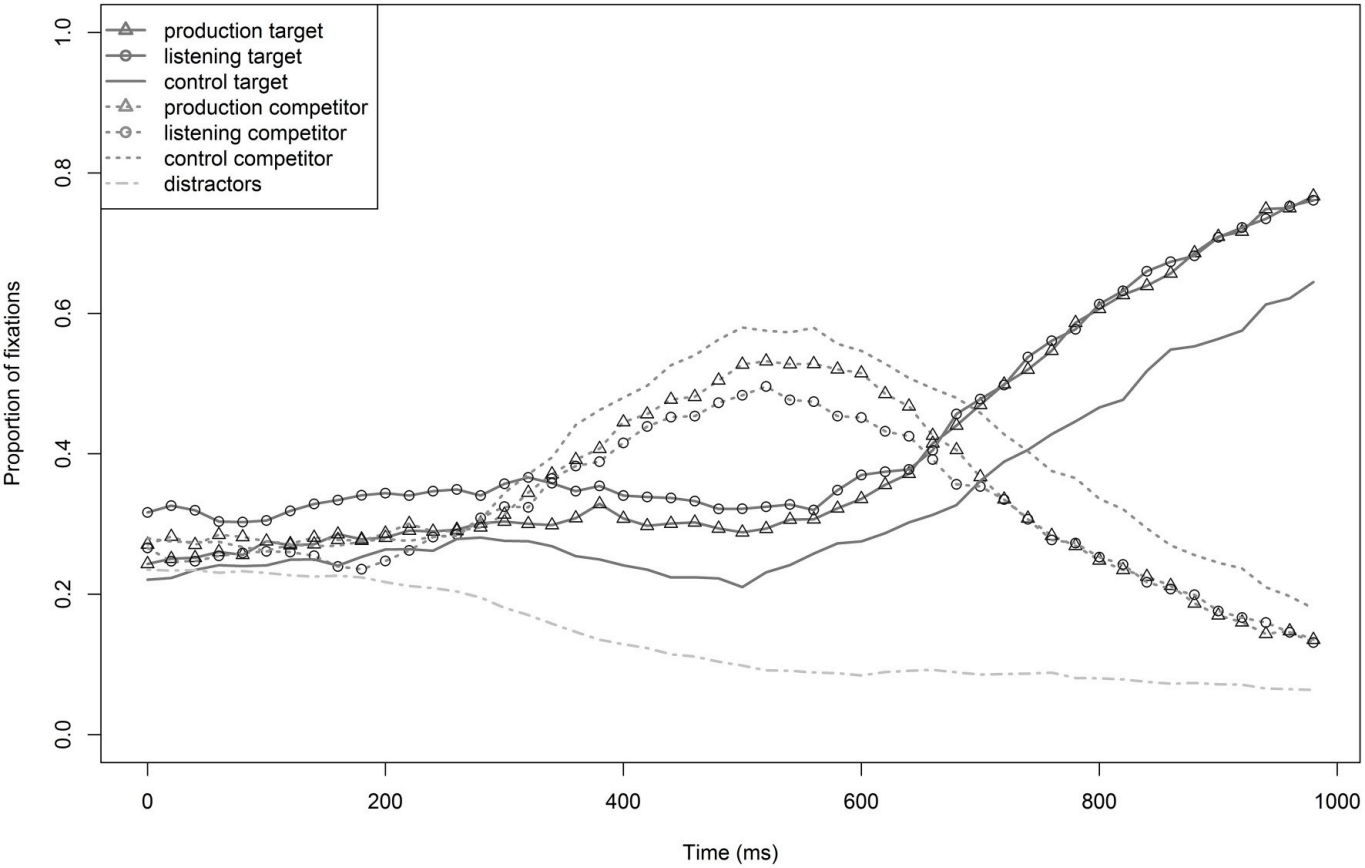
**Proportions of target (*BALKEN*) and competitor (*PALME*) fixations of the production, the listening, and the control group in Experiment 1**. The bottom line describes the mean number of distractor fixations of all three groups.

Not surprisingly, the distractors were ruled out as potential target words very early by the participants (from about 200 ms, see Figure 2), i.e., the fixation proportion of distractors decreased very quickly. As launching a programmed eye movement usually takes about 200 ms (e.g., Altmann and Kamide, 2004), word processing is reflected in fixation proportions from this point in time on. Competitors were preferred over targets by all three groups from about 280 ms on until about 700 ms. Target fixations show that the two training groups started to fixate the target more often than the control group from about 250 ms on. The advantage of both training groups became more pronounced and started being robust from about 350 ms on. Visually, there was no difference between the production and listening groups. Statistical analyses were run for the time window 250–750 ms because it included the whole process of target-competitor disambiguation, and here it became evident that the two training groups had a stable advantage of target fixations compared to the control group. As can be seen in Figure 2, the actual advantage lasted much longer—at least until 1000 ms.

#### Statistical analysis

First, a model with data in the time window 0–200 ms was run. This tested looking biases before processing of the actual target word began. Training group was the fixed effect, and participant and item were random intercepts. There was a significant effect by training (χ^2^ = 7.2, *p* < 0.03); the results of the mixed model show that the listening group had more target fixations than both the control group (ß_*training*_ = 0.39, *SE* = 0.15, *t* = 2.6) and the production group (ß_*training*_ = 0.31, *SE* = 0.15, *t* = 2.1), hinting at a target bias for this group.

The second model analyzed data between 250–750 ms. It included training group and sound condition as fixed effects as well as participant and item as random intercepts. Training was significant (χ^2^ = 10.7, *p* < 0.005); both the listening group (ß_*training*_ = 0.48, *SE* = 0.15, *t* = 3.2) and the production group (ß_*training*_ = 0.33, *SE* = 0.14, *t* = 2.3) fixated the target more often than the control group. There was no difference between the two training groups (*t* = 1.0). Furthermore, there was a main effect of sound condition (χ^2^ = 7.5, *p* < 0.007), resulting in more target fixations for bilabial than velar items (ß_*condition*_ = 0.35, *SE* = 0.13, *t* = 2.8). Due to the bias for the listening group found from 0–200 ms, the critical time window was further examined. On average, from 0–200 ms the proportion of target fixations was 8% higher for the listening group than for the control group. To account for this early bias, we subtracted 8% from listening group data between 250–750 ms and re-ran the same model with the modified data. Despite the reduction of the listening group's target fixation data, training was still significant (χ^2^ = 6.2, *p* < 0.05): the listening group still fixated the target more often than the control group (ß_*training*_ = 0.30, *SE* = 0.15, *t* = 2.0), and there was no difference between the production and listening groups (*t* = 0.2). This suggests robust differences between the control group and the two training groups.

### Discussion

We found that accent adaptation was possible after both listening and production training. The proportion of target looks in both training groups was higher than in the control group. The listening group, however, fixated targets more often than the other groups, even before actual target word processing began, which might be argued to have affected the listening group advantage in the subsequent critical time window. This, however, can be excluded because the pattern of results persisted even when the fixation data of the listening group in the larger, later time window were penalized for its advantage in the initial, smaller time window. There were no effects of speaker, i.e., learning occurred equally well with speaker A and B. The main effect for sound condition may be related to specific sound properties but does not further affect the general pattern of results. Moreover, the same pattern was observed for old tokens from the training phase and new tokens, indicating learning of a rule that generalizes to new words.

Our results suggest accent learning for the production and listening groups, with no difference between the two training groups. Thus, we found robust effects of accent training when testing single accented words, hinting at a great effect by target words' accent as objective criterion of salience. Production and listening training seemingly do not differ from one another for L1 in terms of salience.

Experiment 1 provides evidence for successful accent adaptation after listening to or producing an accent. However, the canonical competitors (*Palme*) were activated for a very long time (until about 700 ms) before the devoiced target word was fixated more often. This time window covers the entire initial portion of the word before disambiguation (average disambiguation point: 280 + 200 ms for launching the eye movement = 480 ms; earliest disambiguation point: 150 + 200 ms = 350 ms; longest disambiguation point: 420 + 200 ms = 620 ms) and even longer. This suggests that, despite successful accent adaptation, canonical word forms still remained more easily accessible than accented word forms. There was potentially not enough accent exposure for the accented forms to be able to fully compete with canonical word forms. We suggest that if a learned accent were to be able to have effects on the access of canonical, voiceless words with the same onset as the accent's target form, a greater amount of training is required.

Experiment 2 examines whether accent learning can be strong enough as to affect the processing of voiceless, canonical words with double the amount of accent training. Successful accent learning could imply competition effects from words that were previously not included as competitors. Thus, accent training has potentially effects not only for understanding accented word forms, but accented forms can function as competitors and affect the recognition of canonical word forms. As opposed to Experiment 1 where highly salient accented target words were tested, in Experiment 2, we focused on test words that are expected to have a much smaller degree of salience based on the objective criterion “accent,” i.e., standard German canonical words. Training effects of devoiced words (^*^Palken) were tested on words that canonically start with the accent's target sound (*Palme*). In order to increase the likelihood that accented forms could influence target recognition in their function as competitors, the training was doubled. If the accent is robustly learned, we would expect fewer target fixations by the training groups than without accent training.

## Experiment 2

Again, three participant groups were tested. The training involved the same tokens as in Experiment 1, but the amount of training with the devoiced tokens was doubled. During test, participants did not hear the devoiced words (^*^Palken) this time, but voiceless, canonical words (*Palme*), while seeing the same printed words on the display as the participants from Experiment 1.

### Participants

Seventy-eight female students from the University of Tübingen participated for monetary reimbursement. Six had to be excluded due to calibration problems, resulting in 72 participants (18–31 years, mean = 23.2, *SD* = 3.2; 14 left-handed) who successfully completed the experiment. None of them suffered from any hearing disorders, all had normal or corrected-to-normal vision, and German was their only mother tongue^4^. The participants were randomly assigned to one of the three experimental groups (24 production, 24 listening, and 24 control group).

### Methods and material

The training list was based on that of Experiment 1. However, devoiced (^*^Palken) items were presented twice in a row (rather than just once), resulting in 100 training trials in total (twice the amount of training with the devoiced tokens compared to Experiment 1). Due to the greater amount of training, the training phase took 1 min longer.

During test, the same word quadruplets were presented on the screen—92 critical trials and 4 practice trials with the same properties as in Experiment 1. However, the roles of target and competitor words were switched. Targets were now voiceless tokens (*Palme*) in their canonical form, and competitors were words that have a voiced onset in their canonical form (*Balken*). Auditorily, voiceless words were presented (*Palme*) that matched in their onset with the target word on the screen (*PALME*). Voiced tokens (*BALKEN*) that had been devoiced during the training (^*^Palken), were visually presented competitors. All target words had already been recorded in the recording session for Experiment 1 by the same female speakers.

### Analysis and results

The same procedure for analysis as in Experiment 1 was applied. During training, the production group performed quite well in accomplishing the substitutions (mean: 0.8 errors out of 56 devoiced word trials). The accuracy of clicks during the test phase was 99.8% (equally distributed across training groups). Saccades (17% of the data) and fixations that did not fall into one of the four interest areas (3%) were removed prior to analysis.

#### Descriptive analysis

**Figure 3:**
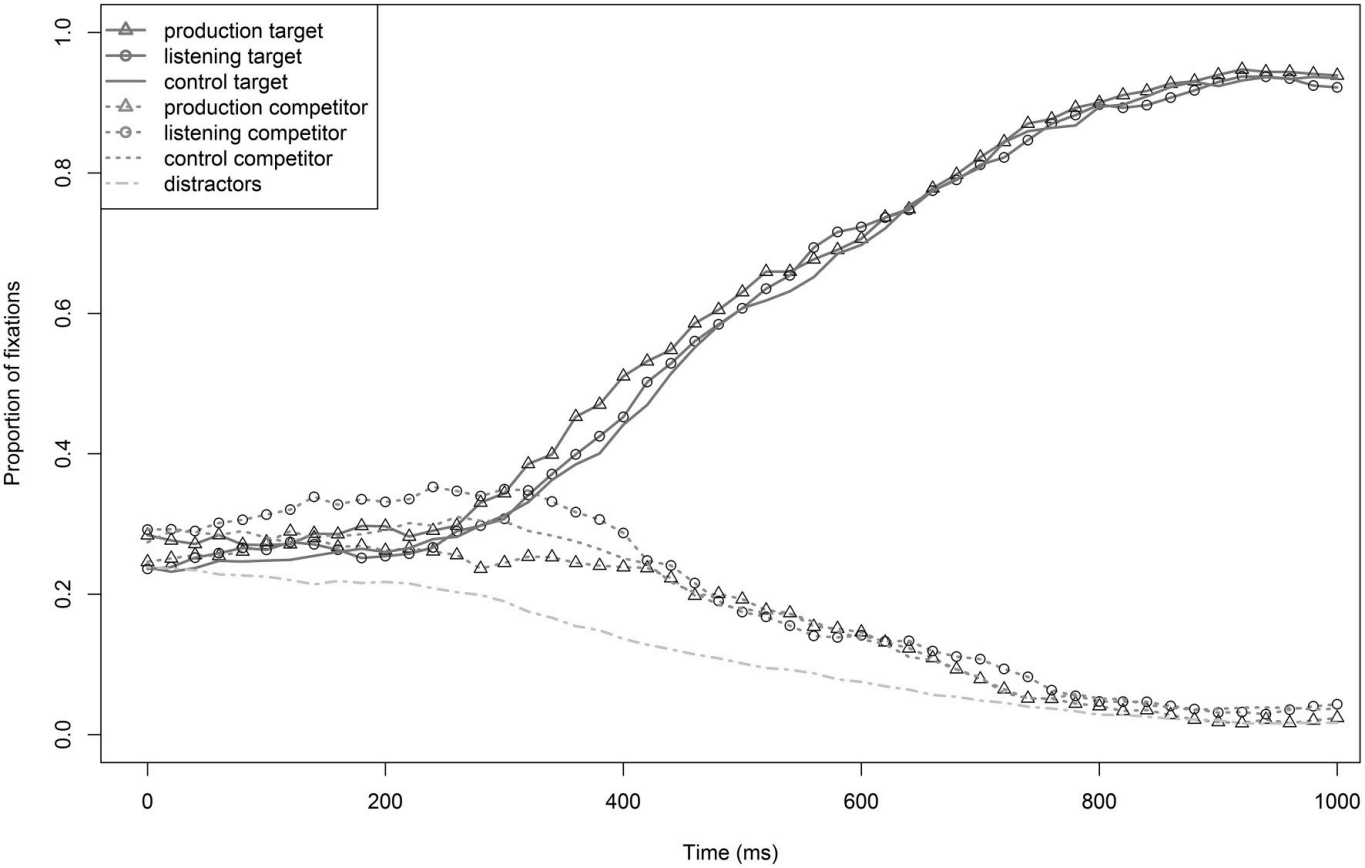
**Proportions of target (*PALME*) and competitor (*BALKEN*) fixations of the production, listening, and control groups in Experiment 2**. The bottom line describes the mean number of distractor fixations of all three groups.

Figure 3 illustrates the proportions of target, competitor, and distractor fixations of the production, listening, and control groups. The distractors were ruled out from the beginning of word processing (200 ms) on, meaning that the proportion of fixations decreased. Target (*PALME*) preference started very early (at about 250 ms), and competitors (*BALKEN*) were quickly ruled out as potential target words. The competitors stayed at a relatively stable level of activation from 200–400 ms, and then fixations decreased noticeably. This represents approximately the interval where target and competitor still overlap (mean overlap: 273 ms). Target fixations by training group did not differ from one another from the beginning until the overall mean end of word processing (measurements of the voiceless target words resulted in a mean word duration of 767 ms). Disambiguation between targets and competitor occurred relatively early, and there was no clear advantage of one of the training groups in target fixations. Statistical analyses were run for the time window from 250–750 ms, which included the entire disambiguation process between targets and competitors and parallels analyses in Experiment 1.

#### Statistical analysis

The baseline model for target fixations (0–200 ms) revealed no significant effect by training (*t* < 1). Mixed effects models revealed no significant effect of any of the considered fixed effects (all *t* < |1.3|) in the critical time window (250–750 ms). Auditorily presented voiceless words (*Palme*) that start with the same onset as the trained, devoiced words (^*^Palken) triggered strong target activation from the beginning of word processing on. There was no effect of learning, neither by the production nor the listening group. In contrast to Experiment 1, the test words did not have the critical accent, but the voiceless paired words with the same sound onset as the devoiced, accented words were tested. As the devoiced training words included a sound substitution, the question is, especially for the production group: How much did the acoustic realizations of the devoiced tokens encountered during training differ from those of the voiceless tokens encountered during test? In other words, did the participants' own productions of the accent differ enough from the productions of the test speaker to prevent generalization across speakers? The missing training effect for both groups reinforces the question of effects of single tokens' acoustic properties. Therefore, acoustic properties of both the training materials and the test materials were analyzed in a next step.

### Acoustic analyses

Pre-recorded target and training stimuli as well as the tokens produced by the production group during training were analyzed acoustically. This tested if the difference between training and test stimuli was too great for adaptation effects to be observed. Particularly in the production group, the acoustic properties of the accented plosives were likely to vary individually. The stops that mark the manipulated accent were focused on in the analysis. Voice onset time (VOT) and burst intensity (relative to total word intensity) were measured for each token that was part of an old critical word pair, i.e., word pairs that were included in both the training and the test phase. Only old word pairs were included in analyses, because there was no reference to the training phase for new words. Each critical voiceless word (*Palme*) and its devoiced paired word (^*^Palken) was considered for analysis. Both instances were taken as separate reference points in order to calculate the differences of the respective acoustic property value between the training and the test token (*Palme*). In the following, we refer to the *Palme-Palme* comparison as the voiceless word pair and the ^*^Palken-Palme comparison as the devoiced word pair. During training, one word was presented several times (devoiced: four times, voiceless: twice). This did not pose any problem for the listening group items because the same recording was presented several times. For the production group, however, single tokens differed individually. This issue was solved by taking average values. Two VOT- and two burst intensity difference values were assigned to each critical word for each participant—one with the values from the voiceless word as a reference point (*Palme*) and one with the values from the devoiced word as a reference point (^*^Palken). Voices differed between training and test in both the listening and the production condition, so minor differences were inevitable.

First, we compared the absolute training-test differences of the acoustic properties of the initial stops [i.e., dif(stop value) = |stop value_Test(Palme)_–stop value_Training(^*^Palken or Palme)_|]. Measurements for all old word pairs were made for VOT (min = 0.14 ms, max = 71.8 ms, mean = 19.9 ms, *SD* = 16.6) and intensity ratio of the burst (min = 0, max = 0.35, mean = 0.08, *SD* = 0.06). These values were compared between the listening and production groups as well as between the devoiced and voiceless training words that included a stop.

**Figure 4:**
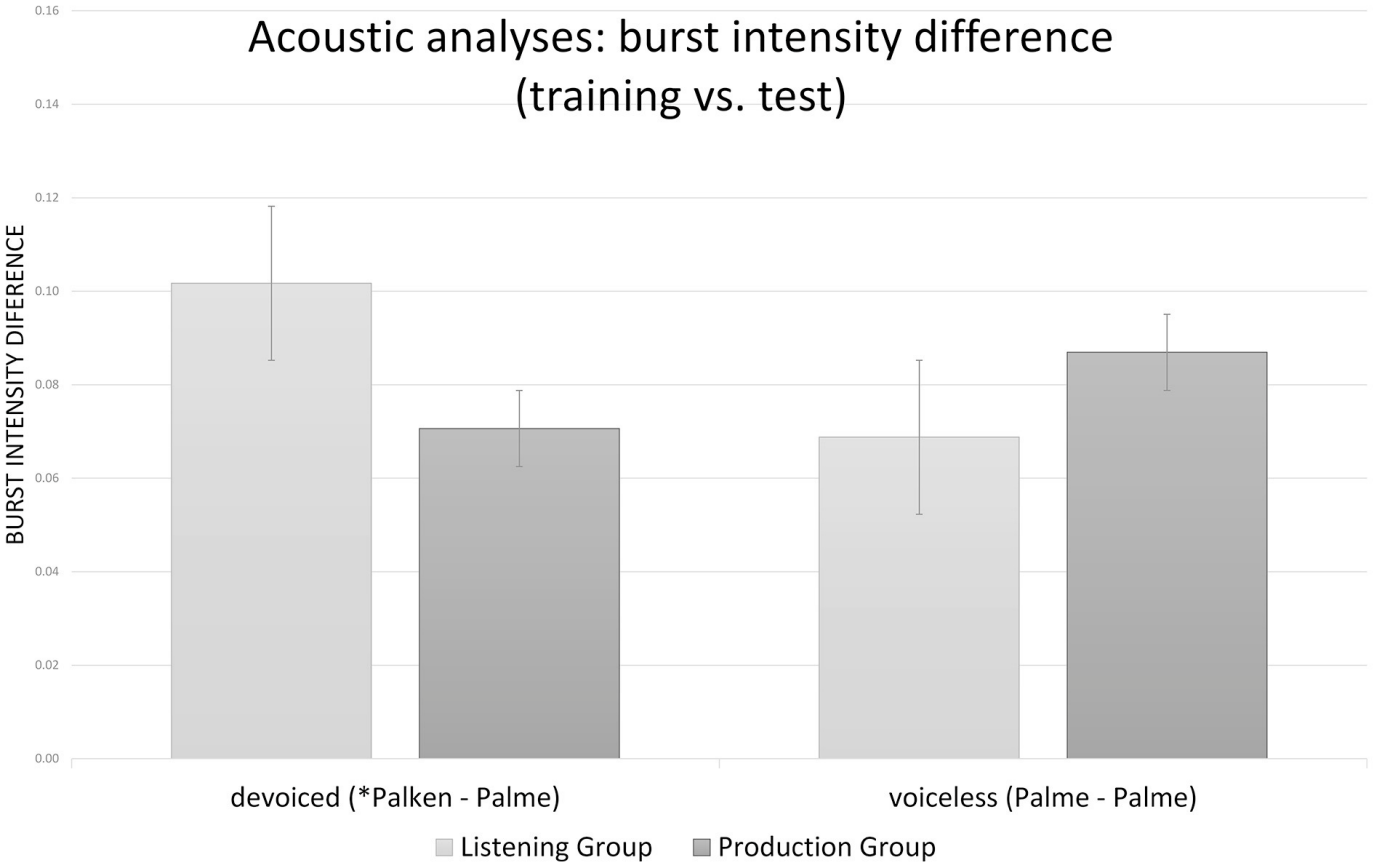
Acoustic differences of relative burst intensity for devoiced (^*^Palken) vs. voiceless (*Palme*) word pairs and listening vs. production training. Whiskers represent standard errors.

For each VOT difference and burst intensity difference mixed effects models were run. Each model included the acoustic variable of interest as the dependent variable. Training (listening vs. production) and word pair (devoiced vs. voiceless) were considered fixed effects, and participant and item were random effects. The model for VOT differences also included by-participant random slopes for training and word pair, as well as by-item random slopes for training. None of the factors resulted in significant effects for VOT difference (all *t* < |0.7|). The model for burst intensity included by-subject random slopes for word pair and by-item random slopes for training. There was a significant interaction between training and word pair (χ^2^ = 5.6, *p* < 0.02) illustrated in Figure 4. Examining the results of the mixed model (see Table 1), this interaction is based on the smaller burst intensity difference for devoiced word pairs in the production group than the listening group (*t* = −2.15), and there was no difference for voiceless word pairs between training groups (*t* = 1.23). Within training groups, there was no training-test difference between devoiced and voiceless word pairs (*t* < 1.8).

**Table 1:** Results for burst intensity differences between training and test words as calculated by the model lmer (burst difference~word pair^*^training + (1 + word pair|participant) + (1 + training|item)).

**Predictor**	**ß**	***SE***	***t***
Intercept (devoiced, listening)	0.10	0.01	7.51
word pair = voiceless	−0.03	0.02	−1.75
**training = production**	−**0.03**	**0.01**	−**2.15**
word pair^*^training	**0.05**	**0.02**	**2.49**
Intercept (voiceless, listening)	0.07	0.01	5.00
word pair = devoiced	0.03	0.02	1.75
training = production	0.02	0.01	1.23
word pair^*^training	−**0.05**	**0.02**	−**2.49**
Intercept (devoiced, production)	0.07	0.01	9.31
word pair = voiceless	0.01	0.01	1.61
**training = listening**	**0.03**	**0.01**	**2.15**
word pair^*^training	−**0.05**	**0.02**	−**2.49**

*The factors were releveled in order to calculate the model with different intercepts. This allows displaying t-values for all relevant level comparisons. β = Estimate, SE = Standard Error. Bold levels are significantly different from the intercept (t>|2|)*.

### Discussion

Neither training group fixated the target less often than the control group without training did. They did not differ from one another in their amount of target fixations. The recognition of voiceless *Palme* was not affected by previously having learned that *Balken* is pronounced as ^*^Palken. This occurred despite the fact that accent training was intensified by presenting devoiced tokens twice as often as in Experiment 1. This is good news for native accent listeners, because it shows that learning a new accent does not immediately distort comprehension of canonical forms. Concrete acoustic analyses tested whether this effect was due to greater inherent salience based on an objective criterion of devoiced (as tested in Experiment 1) compared to voiceless tokens or rather because of greater acoustic differences between the devoiced training and the voiceless test tokens. There was no VOT difference between training groups, thus the production group was quite good at accomplishing the substitutions. The few production errors that occurred did not affect the overall pattern. This was also supported by the observation that burst intensity differences were even smaller for the production group than the listening group.

## General discussion

The present study investigated whether different forms of native accent training and different token realizations (unaccented vs. accented) differ in salience for L1 participants. This was measured by the amount of adaptation to the native accent. As a subjective criterion of salience, the training phase was varied by having production and listening accent training (vs. no training), and an objective criterion was tested by the nature of the test tokens (accented/devoiced words in Experiment 1 vs. canonical/voiceless words in Experiment 2). In Experiment 1, native German participants produced or listened to single German words that featured the devoicing of initial voiced stops (*Balken* pronounced as ^*^Palken). In the subsequent eye-tracking task, participants from both training groups fixated the devoiced target more often than participants without training did, with no difference between the two training groups. This was true whether the accented target word had been included in the preceding training or if it was presented for the first time. Experiment 2 started with the same accent training and in the test standard German canonical words with the same onset as the devoiced tokens (*Palme*) were targets. The proportion of target looks was not affected by training. Acoustic analyses showed that devoiced training words (^*^Palken) and voiceless test words (*Palme*) did not differ strongly in their onset.

### Salience and adaptation

In Experiment 1, there were significantly more looks to devoiced targets after production and listening accent training than without training. In Experiment 2, which featured voiceless target words, target looks did not reveal accent adaptation. This can be explained by the role of salience in accent adaptation. Two criteria for salience were manipulated and tested in our study. First, an objective criterion was tested through the nature of the test tokens (accented/devoiced test words in Experiment 1, canonical/voiceless test words in Experiment 2). The devoiced test words were predicted to be more salient than the voiceless words, thereby resulting in greater adaptation effects. Second, a subjective criterion was tested by having an accent training session based on different modalities (production and listening).

In terms of the objective criterion, adaptation only showed effects for devoiced, and not for voiceless, target words that had the same word onset (^*^Palken vs. *Palme*). This suggests that devoiced tokens are more salient than voiceless tokens. Acoustic analyses of Experiment 2 support our interpretation. There was no evident acoustic difference between devoiced and voiceless word onsets that could have inhibited learning. Training was still effective, though not visible, because the test tokens were not as salient as in Experiment 1. Test tokens in Experiment 1 and 2 therefore only differ perceptually from their disambiguation point onward (after /pal/ for ^*^Palken and *Palme*). This implies that training effects emerged only in later stages of processing, after word disambiguation. This is supported by the analysis in Experiment 1, where the training group advantage admittedly was already detectable from about 250 ms on (see Figure 2); however, the plot of fixation proportions suggests that the two training groups' advantage increased over time and became stable from about 350 ms on. The shortest duration of the ambiguous word section (i.e., overlapping with the competitor) measured approximately 150 ms in Experiment 1 (*ger* in *Germane* “*Teuton*,” speaker A). The moment where the information after the disambiguation point is processed is then reflected from about 350 ms after the stimulus onset onward (150 + 200 ms eye movement launching). Cho and Feldman (2013) found a memory advantage for accented compared to canonical words. They argue that accented speech is more variable in terms of acoustic and phonetic detail, and, based on an episodic account of the mental lexicon, they suggest that difference between accented speech input and stored exemplars is greater than the difference between unaccented input and stored exemplars. Accordingly, this greater difference enriches the form-meaning relationship. This reasoning essentially follows the same principles as the distinctiveness account of salience. More distinct tokens are more salient, which results in memory advantages. It can be argued that salience of accented tokens in the present study was artificially increased by the fact that there was only one specific accent marker and no more natural, global accent. However, a cross-modal priming study by Eisner et al. (2013) found that L1 English listeners adapt to final devoicing in English (*seed*, pronounced [si:t^h^]) when it was produced either by a native British English speaker or by a native Dutch speaker with L2 English (with global Dutch accent features). Moreover, the findings from the Cho and Feldman study are in line with ours. They incorporated a global accent (Dutch-accented English) and still found a memory advantage of accented over canonical tokens.

A subjective criterion of salience, on the other hand, was implemented through the training session. The production group was compared to the listening group as well as the control group without training. Accent adaptation worked equally well with both listening and production training in Experiment 1 (target ^*^Palken), and effects were not visible with voiceless (*Palme*) targets in Experiment 2. There was no difference between the two training groups in either experiment. This suggests that both production and listening accent training imply a similar amount of salience in the fostering of accent adaptation, and adaptation effects become visible only when the test token receives sufficient salience through an objective criterion.

Interestingly, we found that in L1, salience elicited by the subjective criterion of producing an accent was as large as that of listening to the accent. In a previous study (Grohe and Weber, in press), the effects of production vs. listening training on accent adaptation were tested for both L1 and L2 participants. L2 participants adapted to the accent most easily with production training. L1 participants did not adapt, neither with listening nor production training. Importantly, all speech in the present study was produced by L1 speakers, but in Grohe and Weber, test items were always produced by an L2 speaker of English. Thus, for L1 participants in the production training group there was a switch in nativenesss of the speaker between training (L1) and test (L2). L2 speakers likely involve a greater amount of variability (Wade et al., 2007) in their speech than L1 speakers, including more accent markers which probably require additional processing. Moreover, the similarity between listener and speaker is emphasized by the *integrated theory of language comprehension and production* (Pickering and Garrod, 2013), according to which a listener's previous production experience can affect comprehension. This experience is predicted to have greater effects with increasing speaker-listener similarity. The present results, however, do not necessarily support this suggestion. In spite of greater speaker-listener similarity (same sex, same L1 background, mostly similar dialects), the production group did not have greater training effects than the listening group. Nevertheless, having an L1, not L2, speaker produce the accent helped L1 participants to adapt to an accent after both listening and production training. Contrary to L2 participants in Grohe and Weber, however, accent adaptation was not stronger after production training. Producing an accent is only a more important subjective criterion of salience than listening, because of specific L2 properties (e.g., greater perceptual flexibility). There is no general advantage exhibited by producing compared to listening.

Taken together, there was arguably no advantage of production over listening training for L1 listeners, because production might only make a linguistic token more salient if it can act as objective, not subjective, criterion of salience. This would additionally include that the concrete situation determines salience. Furthermore, the studies that have found robust production effects (MacLeod, 2011; Cho and Feldman, 2013) were all memory studies that tested active and conscious word recall, thus later stages of processing. Contrarily, the present eye-tracking study tested online word processing. It is therefore also possible that the production advantage may not arise in the earliest stages of processing. Other studies conducted a repetition experiment rather than a listening-only task as we did (e.g., Cho and Feldman; Kartushina et al., 2015). Repetition includes listening and producing the critical token, possibly implying a greater amount of salience than only production. Finally, concrete feedback may have affected the results of the study by Kartushina and colleagues.

Referring again to the definition of salience established in the beginning of this article, MacLeod (2011) suggests that for mixed lists (including items both listened to and produced), produced items are more distinct and therefore more salient. This kind of salience likely relies on an objective criterion—the stimulus itself attracts attention because of its distinct physical characteristics. In the present study, on the other hand, it was asked if the nature of training (production vs. listening) could act as a subjective criterion of salience. Our results do not support a production advantage *per se*, but they also do not exclude the possibility of a production advantage. The production advantage may function within the scope of salience that relies on an objective, but not subjective, criterion, even with L1 participants and in an online task. In summary, we have found salience effects that rely on an objective criterion and no effects that rely on a subjective criterion. Previous studies that support the production effect have always tested salience arising from an objective criterion. We hypothesize that the nature of salience is the crucial factor in the adaptation process and that, in short-term adaptation, objective criteria are more powerful than subjective criteria.

This contrasts at first sight with findings on dialect accommodation by Auer et al. (1998), who emphasize the importance of subjective criteria of salience. Note, however, that the researchers refer to change in production over the long term rather than to comprehension in the short term, as was tested in the present study. Therefore, different criteria of salience might result in salience that is most efficient at different stages of adaptation and in different modalities. On the other hand, these results are good news regarding short-term comprehension adaptation in language change contexts. These contexts mostly involve new and old pronunciation variants, resulting in contrasts between the two. This provides well-suited conditions for an objective criterion of salience in terms of contrasts in phonetic realizations. Adaptation will be easier in contact situations than it would be in potential accent-only situations. At the same time, as accent comprehension improves, comprehension abilities of the canonical pronunciation are not impaired. If we apply our results and those from Grohe and Weber (in press) to concrete L2 learning situations, we can conclude that for learning new variations, L2 learners, thanks to their greater cognitive flexibility, can still achieve reasonable results without switching between production and listening. It would, however, probably be even more beneficial to integrate variation between the two modalities.

### Competitor inclusion as a further step in adaptation

Adaptation was observed not only for old words that had been part of the training phase; it also generalized to new words with the same accent and furthermore from the voice of the training speaker to the unfamiliar voice of the test speaker. This finding supports abstractionist accounts of the mental lexicon (McClelland and Elman, 1986; Norris, 1994) rather than episodic accounts (e.g., Goldinger, 1998). Whereas episodic accounts suggest the storage of every concrete exemplar of a speech unit encountered by a listener (including speaker-inherent details, e.g., voice properties), in abstractionist models, abstract representations of a word's canonical representation build the lexicon. Variations of the canonical form, such as accents, can be accounted for by pre-lexical mapping rules. These rules are built on the basis of a few exemplars that are no longer stored. When, for example, an accented token is encountered after accent training, the learned rule is applied to the respective abstract entry in the lexicon. This can explain why learning a specific variation can generalize across many different words (McQueen et al., 2006). However, we do not want to rule out the existence of exemplars in the lexicon. Hybrid models (e.g., McLennan et al., 2003) attempt to integrate exemplars and pre-lexical rules into a single account. In contrast to Bradlow and Bent (2008) and Sidaras et al. (2009), who observed speaker generalization only if training was conducted with multiple voices, one voice was sufficient for generalization in Experiment 1. The globally accented speakers in those studies likely featured many different accent markers, resulting in a stronger accent than the accent we presented. With only few accent markers, it is easy to build pre-lexical accent rules allowing for generalization to new talkers. With many different accent markers, however, multiple exemplars from multiple talkers might help successful rule-building as argued by Bradlow and Bent.

Moreover, Trude and Brown-Schmidt (2012) tested competitor exclusion and inclusion and found talker-specific adaptation effects. Competitor exclusion and inclusion describes modifications in the cohort of words initially activated when a word starts to be processed. Potential candidates can be excluded, or new candidates can be added (competitor inclusion). Effects of accent training on competitor activation are indirect training effects—the effects of the accent on other words (presented as targets) are then tested. These tokens seem less salient than accented tokens. It seems that if less salient targets are tested, the role of aspects such as talker specificity increases. In other cases, these aspects may be training intensity or prior accent familiarity, as shown in Trude et al. (2013). The design of their study was similar to the eye-tracking study discussed above (Trude and Brown-Schmidt, 2012). Talker-specific effects of accent learning on competitor exclusion were again tested, but this time with a Québec-French accent that participants had never been exposed to before the experiment. The talker replaced every /i/ with an /I/ in English words, i.e., *weak* became *wick*. An accent training session did not help participants rule out unlearned competitors more easily if pronounced by the accented talker than the unaccented talker. As suggested by Trude et al. (2013), competitor exclusion failed seemingly due to the accent being completely new to participants. Considering the small amount of target word salience, more previous accent exposure (as shown in Trude and Brown-Schmidt, 2012) or greater training intensity could have helped. This interpretation is also supported by Experiment 2 of the present study. In contrast to the accented, devoiced targets from Experiment 1, the canonical, voiceless targets in Experiment 2 implied smaller overall objectively induced salience. Additionally, the accent was completely new to the participants.

We found that after a few minutes of training, an accent can be learned so that it is more easily processed than without training. Only highly salient target tokens made learning effects visible. Therefore, accent training does not always exhibit robust accent learning. As shown by Trude and Brown-Schmidt (2012), this does not mean that more robust accent learning is not impossible. They found effects of both competitor exclusion and inclusion when non-salient target tokens were tested. The effect was talker-specific, and the participants already had prior (pre-experimental) experience with the accent. Accent adaptation seems to occur in various steps, ranging from unadapted to partially adapted (effects can be observed for accented, salient words) all the way to fully adapted (effects can be observed for unaccented, non-salient words). Full accent adaptation would mean that the way that accented word forms function as competitors is similar to the functioning of canonical word forms. However, the amount of looks to the targets in Experiment 2 was the same with and without training, indicating that full adaptation had not occurred. It likely requires more intense training, pre-experimental accent familiarity, identical talkers during training and test, or even multiple talkers during training (Bradlow and Bent, 2008). The adaptation effects that we found seem to reflect partial accent adaptation, which is still important because it allows a listener to better understand the accented form itself. The reason why we did not find full adaptation can also lie in the native language background of our listeners. Bent and Bradlow (2003) found that non-native listeners performed equally well in a sentence recognition task while listening to a speaker with the same L1 as when listening to a native speaker. This advantage has even generalized to unrelated accents that were new to the listener. Native listeners, on the other hand, as shown in a training-test study by Baese-Berk et al. (2013), are only able to generalize accent learning to a new accent if they are trained on many different accents. This finding is in line with the results on generalization of accent learning to new voices by Bradlow and Bent (2008).

Basic assumptions from abstractionist accounts on lexical processing support our conclusion that the accent rule was not learned strongly enough to be applied to all tokens from word onset onward. In Experiment 1, the voiceless competitors (*PALME*) of the target word ^*^Palken were considered as potential candidates for a long period, and only after disambiguation was the target *BALKEN* fixated more often than the competitor. With the auditory target *Palme* in Experiment 2, the pre-lexical rule was not learned strongly enough to establish additional competition by *BALKEN* during the /pal/-segment. One could assume that the results of Experiment 2 are due to increased competitor (*BALKEN*) activation. Participants learned that *Balken* becomes ^*^Palken, so they might have concluded that *Palme* becomes ^*^Balme. This is rather unlikely, however, because the training also included canonical words starting with the voiceless sound (*Palme*). Therefore, when hearing *Palme*, they did not interpret the word input as ^*^Balme and thus did not fixate the competitor more often than the target.

### Native and foreign accent adaptation

In our discussion, we included studies that tested adaptation to native accents as well as studies on adaptation to foreign accents. Research on foreign accent adaptation clearly shows that in their L1, listeners quickly adapt to foreign accents produced by L2 speakers and maintain long-lasting processing advantages (e.g., Clarke and Garrett, 2004; Maye et al., 2008; Witteman et al., 2013, 2015). Similar findings arise from native accent studies (Trude and Brown-Schmidt, 2012). It is therefore possible that a dichotomy between native and foreign accents is unjustified. Similar mechanisms could apply to both native and foreign accent processing. Clarke and Garrett (2004) suggested an accent processing classification that depend on the accent's acoustic distance from native speech. Foreign and native accents follow the same principles, but the strength of an accent could determine the nature of accent adaptation. Arguably, native accents can be closer to standard native speech than foreign accents. Processing of regional and foreign accents could then rely on similar mechanisms, but stronger accents induce greater processing effort than mild accents do. As a consequence, adaptation to regional accents would be easier than adaptation to foreign accents. This account would explain why, on the one hand, similar results were found if the same accent was produced by an L2 or L1 talker (Trude et al., 2013), and, on the other hand, greater processing difficulties were found for foreign than for native accents (Floccia et al., 2006, 2009). Likewise, we found adaptation for L1 participants when an L1 speaker produced the contrived accent in the present study, but in a previous study (Grohe and Weber, in press), adaptation was not found when an L2 speaker produced the accent. We suggest that accent strength is very likely linked with the amount of different accent markers that a speaker produces, which varies among individuals. Some L2 talkers do not exhibit many accent features, whereas others do. Therefore, concrete acoustic features could be an important variable which the magnitude of accent learning depends upon.

## Conclusion

In conclusion, our study suggests that native accent adaptation can be fast and easy, including generalization to new voices and new lexical tokens as well as learning through individual production. However, the accent requires salience that relies on an objective criterion during test in order to display its adaptation effects. The strength of accent learning is therefore limited; an accent is not learned well enough to affect the processing of other, non-salient canonical tokens. It is not integrated as strongly into the lexicon as canonical tokens. Learning was not affected by our training manipulation, which relied on a subjective criterion of salience. There are, however, studies that have found an advantage of production over listening when training functioned as objective criterion of salience. We therefore conclude that in short-term accent adaptation listeners might be more sensitive to objective than to subjective criteria of salience.

## Ethic statement

This study was carried out in accordance with the recommendations for ethical guidelines of the English Department (Psycholinguistics and Applied Language Studies), University of Tübingen, Germany. All participants gave informed consent in accordance with the Declaration of Helsinki.

## Author contributions

All authors listed have made substantial, direct and intellectual contribution to the work and approved it for publication by agreeing to be accountable for all aspects of the work. Concretely, AG and AW developed the conception of the study based on prior findings and created the experimental design, discussed stimuli, interpreted results and revised prior versions of the manuscript together. Moreover, AG compiled and recorded experimental stimuli, ran participants, processed the data, conducted statistical analyses, and wrote the first draft of the manuscript.

### Conflict of interest statement

The authors declare that the research was conducted in the absence of any commercial or financial relationships that could be construed as a potential conflict of interest.

## Footnotes

^1^For example, in their account on social weighing in speech perception, Sumner et al. (2013), predict better memory for accented words—but only if the accent is socially prestigious.

^2^Fifty participants indicated dialect familiarity (42 specifically with a Southern German dialect, mostly Swabian). As most dialect speakers had exposure to a Southern German dialect, the variable “Southern Dialect” was tested in initial data analyses, resulting in no significant effect. Participants were not selected based on dialect competence, and it was not counter-balanced across conditions; therefore this factor was not included in the methods section.

^3^In some varieties of German, speakers tend to devoice initial voiceless stops when they are followed by a liquid (e.g., *grillen* can be pronounced as *krillen*). By always having a vowel following the initial consonant, potential previous experience with the accent was avoided.

^4^Fifty-three participants indicated dialect familiarity, 50 of whom had exposure to a Southern German dialect (mostly Swabian). The variable “Southern Dialect” was tested in initial data analyses, resulting in no significant effect, as in Experiment 1.

## Appendix

**Table A1:** **Critical target-competitor pairs for Experiments 1 and 2**.

	**Voiced**			**Voiceless**		
	**German token**	**IPA transcript**	**English translation**	**German token**	**IPA transcript**	**English translation**
bilabial	Butter	ˈ**bʊt**ɐ	butter	Putzer	ˈ**pʊt**sɐ	cleaner
	Bistum	ˈ**bɪst**u:m	diocese	Piste	ˈ**pɪst**ə	ski slope
	Beize	ˈ**bait**sə	marinade/stain	Peitsche	ˈ**pait**ʃə	whip
	Beifall	ˈ**bai**fal	acclaim	Peiler	ˈ**pai**lɐ	detector
	Baron	**baˈr**o:n	baron	Paris	**paˈr**i:s	Paris
	Balken	ˈ**bal**kn̩	beam	Palme	ˈ**pal**mə	palm tree
	Becher	ˈ**bɛç**ɐ	beaker/mug	Pächter	ˈ**pɛç**tɐ	tenant
	Benzin	**bɛn**ˈtsi:n	gas	Pension	**pɛn**ˈzi̯o:n	guest house/pension
	Bilanz	**biˈl**ants	balance	Pilot	**piˈl**o:t	pilot
	Ballett	**baˈl**ɛt	ballet	Palast	**paˈl**ast	palace
	Banner	ˈ**ban**ɐ	banner	Panne	ˈ**pan**ə	breakdown
	Bazille	**baˈ**ʦɪlə	bacillus	Pazifik	**paˈts**i:fɪk	Pacific
	Bettler	ˈ**bɛt**lɐ	beggar	Petzer	ˈ**pɛt**sɐ	telltale
	Bauwerk	**bau**vɛrk	building	Pause	ˈ**pau**zə	break
velar	Gorilla	**goˈr**ɪla	gorilla	Korea	**koˈr**e:a	Korea
	Gulasch	ˈ**gʊl**aʃ	goulash	Kuli	ˈ**kʊl**i	ballpoint pen
	Galerie	**gal**əˈri:	gallery	Kalorie	**kal**oˈri:	calorie
	Gasthaus	ˈ**gast**haus	guest house	Kasten	ˈ**kast**n̩	box
	Gürtel	ˈ**gʏrtl̩**	belt	Kürzung	ˈ**kʏrt**sʊŋ	abridgement
	Gitter	ˈ**gɪtɐ**	grid/fencing	Kittel	ˈ**kɪtl̩**	tunic
	Ganove	**gaˈno:və**	crook	Kanone	**kaˈno**:nə	cannon/rod
	Gammler	ˈ**gamlɐ**	loafer	Kammer	ˈ**kam**ɐ	small room/chamber
	Germane	**gɛr**ˈma:nə	Teuton	Keramik	**kɛˈr**a:mɪk	ceramics
	Geltung	**ˈɡɛlt**ʊŋ	validity/prestige	Kälte	**ˈkɛlt**ə	cold
	Garant	**ɡaˈra**nt	guarantor	Karat	**kaˈra**:t	carat
	Garage	**gaˈra**:ʒə	garage	Karaffe	**kaˈra**fə	carafe
	Gassenjunge	ˈ**gasən**ˈjʊŋə	street urchin	Kassenzettel	ˈ**kasən**ˈtsɛt**l̩**	(sales) receipt
	Gartenzaun	ˈ**gart**n̩tsaun	garden fence	Kartenspiel	ˈ**kart**nʃpi:l	game of cards

Voiced items were used as targets in Experiment 1 (initial plosive was devoiced, i.e., /b//p/ and /g//k/), and voiceless items were competitors. In Experiment 2, voiceless items were targets, and voiced items were competitors.
